# CNX:FAM134B-driven ERLAD of ATZ polymers proceeds via enhanced formation of VAPA:ORP1L:RAB7 contact sites between ER and endolysosomes

**DOI:** 10.1080/27694127.2025.2574355

**Published:** 2025-10-30

**Authors:** Elisa Fasana, Ilaria Fregno, Maurizio Molinari

**Affiliations:** a Faculty of Biomedical Sciences Università della Svizzera Italiana (USI), Institute for Research in Biomedicine, Bellinzona, Switzerland; b School of Life Sciences, École Polytechnique Fédérale de Lausanne, Lausanne, Switzerland

**Keywords:** Calnexin (CNX), Endoplasmic Reticulum (ER), endolysosomes, ER-phagy, ER-to-Lysosome-Associated Degradation (ERLAD), FAM134B, Membrane Contact Sites (MCS), ORP1L, RAB7, VAPA

## Abstract

Membrane contact sites (MCS) between organelles maintain the proximity required for controlled exchange of small molecules and ions yet preventing fusion events that would compromise organelles’ identity and integrity. Here, by investigating the intracellular fate of the disease-causing Z-variant of alpha1 antitrypsin (ATZ), we report on a novel function of MCS between the endoplasmic reticulum (ER) and RAB7/LAMP1-positive endolysosomes in ER-to-lysosome-associated degradation (ERLAD). For this function, the VAPA:ORP1L:RAB7 multi-protein complex forming MCS between the ER and endolysosomes engages, in an ERLAD client-driven manner, the misfolded protein segregation complex formed by the lectin chaperone calnexin (CNX), the ER-phagy receptor FAM134B, and the ubiquitin-like protein LC3. Generation of this supramolecular complex facilitates the membrane fusion events regulated by the SNARE proteins STX17 and VAMP8 that ensure efficient delivery of ATZ polymers from their site of generation, the ER, to the site of their intracellular clearance, the degradative RAB7/LAMP1-positive endolysosomes.

## Introduction

Proteins that fail to attain the native structure in the ER are retro-translocated into the cytosol for proteasomal clearance by ERAD pathways that have been studied in detail in the last 35 years^[1–3]^. An increasing number of disease-causing misfolded proteins that cannot engage the ERAD machinery have been described in recent years. Often, these are large or aggregated polypeptides that fail to be retro-translocated across the ER membrane. Rather, they are segregated in ER subdomains that eventually deliver their toxic content to endolysosomal degradative compartments via ERLAD, an acronym that we introduced to define all autophagic and non-autophagic pathways that ensure the lysosomal clearance of ERAD-resistant misfolded polypeptides from the ER^[4–8]^. ATZ is a model ERLAD client^[9–12]^. An aspartic acid to lysine (E342K) point mutation enhances polymerogenicity of this disease-causing polypeptide^[13]^. ATZ polymers fail dislocation across the ER membrane for ERAD. They undergo repeated cycles of de-glucosylation and re-glucosylation of the ATZ N-glycan at position 83 by ER-resident α-glucosidase II and UDP-glucose:glycoprotein glucosyltransferase 1 (UGT1), which prolong the association of ATZ polymers with the lectin chaperone CNX^[9]^. ATZ:CNX complexes eventually engage the ER-phagy membrane receptor FAM134B and lipidated LC3 to favor segregation of polymeric ATZ in ER subdomains and/or in ER-derived vesicles that release their toxic content within RAB7/LAMP1-positive endolysosomes upon STX17:VAMP8-driven fusion of ER and endolysosomal membranes^[9,10]^. How ER subdomains and/or ER-derived vesicles containing ATZ polymers are docking to the appropriate degradative compartment to deliver their toxic content for clearance is not known.

Membrane contact sites (MCS) are sites of proximity between the membrane of organelles. They are stabilized by tethering machineries that maintain organelles’ proximity but prevent membrane fusion events that would compromise organelle identity and integrity^[14,15]^. The MCS between ER and endolysosomes are well characterized, are maintained by VAPA-ORP1L-RAB7 multiprotein complexes, and serve as site of lipids and calcium transfer^[14–21]^.

**Figure 1.**
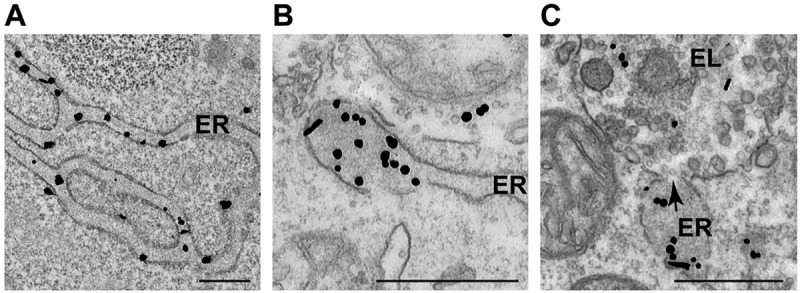
Segregation and lysosomal delivery of ATZ polymers. (A) RT-TEM micrograph showing immunogold-labeled ATZ polymers dispersed in the endoplasmic reticulum (ER) lumen. (B) ATZ polymers are segregated in terminal portions of the ER. (C) The arrow shows the site, where the membrane of the ER subdomain fuses with the membrane of the endolysosomal degradative compartment (EL). Scale bar: 500 nm.

The finding that VAPA is a major interactor of FAM134B^[22]^, as well as transmission electron microscopy (TEM) micrographs showing the proximity of ER subdomains containing ATZ polymers and endolysosomes before and during delivery of the ERLAD client within the degradative compartment (Figure 1)^[10]^, led us to examine if the generation of contact sites is required for lysosomal protein clearance from the ER. Here, we show that cells producing ATZ polymers, i.e., misfolded polypeptides to be selected for lysosomal clearance (i.e., ERLAD), display higher number of ER-endolysosomes MCS compared to cells that do not express misfolded proteins, or that express the NHK variant of alpha1 antitrypsin, a folding-defective polypeptide that is efficiently retro-translocated across the ER membrane for proteasomal degradation (ERAD)^[12,23–25]^. We report that the *segregation complex* formed at the ER membrane by the lectin chaperone CNX and the ER-phagy receptor FAM134B and stabilized upon intralumenal expression of ATZ polymers^[10]^ also contains the ER membrane protein VAPA, the cytoplasmic bridging protein ORP1L and the small RAB7 GTPase at the limiting membrane of the endolysosomes. This generates ER-endolysosome MCS that facilitate delivery of the ERLAD client within degradative compartments upon membrane fusion events controlled by the SNARE proteins STX17 and VAMP8. Inhibiting the formation of ER-endolysosomes MCS upon genome editing to delete the VAPA or the ORP1L proteins substantially hampers lysosomal clearance of misfolded ATZ.

## Results

### ATZ is delivered from the ER lumen into endolysosomes for clearance

Room Temperature (RT)-TEM has been used to monitor the fate of disease-causing ATZ polymers generated in the ER lumen (Figure 1(A), ER), and segregated in ER subdomains (Figure 1(B)) that eventually fuse with endolysosomes (EL) to deliver their toxic content for clearance (arrow in Figure 1(C))^[9,10]^.

**Figure 2.**
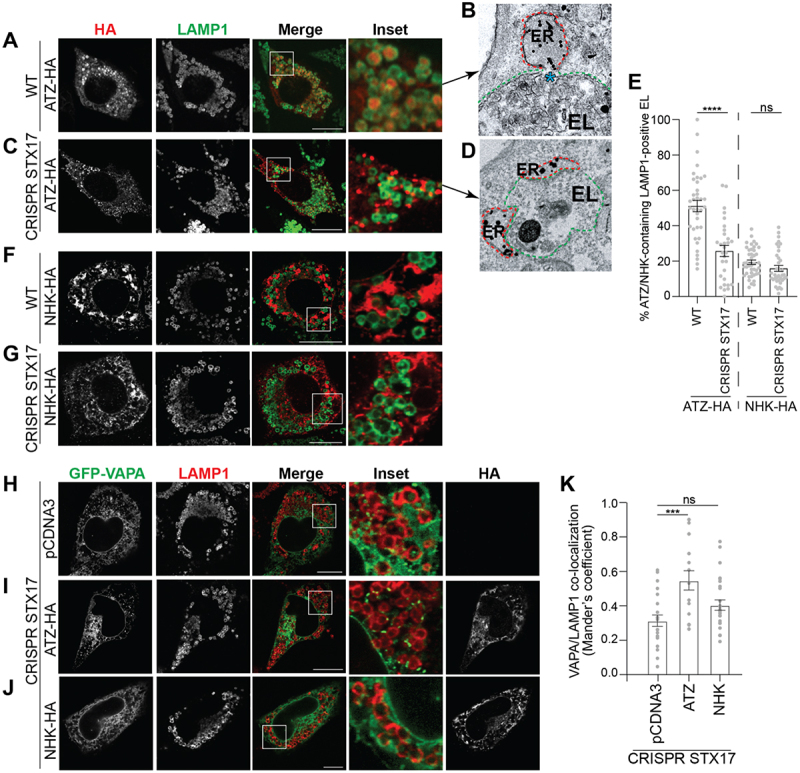
ATZ expression increases ER-endolysosome contact sites. (A) CLSM analyses of ATZ-HA delivery (and accumulation) within LAMP1-positive endolysosomes in wild type MEF treated with 50 nM BafA1. (B) same as a in RT-IEM. The blue asterisk shows the site of fusion between ATZ-enriched ER subdomains (dotted red line) and the degradative organelle (dotted green line). (C) accumulation of ER containing ATZ-HA in proximity of LAMP1-positive endolysosomes in CRISPR STX17 MEF treated with 50 nM BafA1. (D) same as C in RT-IEM. (E) LysoQuant quantification of ATZ delivery within LAMP1-positive endolysosomes in A, C, F, G (*n* = 37, 30, 42, and 36 cells of three independent experiments, respectively). Unpaired two‐tailed *t*‐test, ^ns^*P* > 0.05, ****p < 0.0001. *t* = 5.605 and *t* = 1.686, respectively. (F) NHK-HA does not accumulate within LAMP1-positive endolysosomes in wild type MEF treated with 50 nM BafA1. (G) NHK-HA distribution in CRISPR STX17 MEF treated with 50 nM BafA1. (H) CLSM analysis showing GFP-VAPA and LAMP1 distribution in CRISPR STX17 MEFs mock-transfected, (I) expressing ATZ-HA, or (J) expressing NHK-HA. (K) Mander’s coefficient of VAPA/LAMP1 co-localization of H-J (*n* = 22, 19 and 25 cells of 3 independent experiments, respectively). One‐way ANOVA and Dunnett’s multiple comparisons test, ^ns^*P* > 0.05, ***P < 0.001. F = 5.825. Each data point shows the level of co-localization between VAPA and LAMP1. 1.0, complete juxtaposition of the two channels. 0.0, no juxtaposition. Scale bar: 10 µm.

Inactivation of lysosomal hydrolases upon exposure of mouse embryonic fibroblasts (MEF) to Bafilomycin A1 (BafA1) results in the accumulation of undegraded ATZ polymers that have been delivered within RAB7/LAMP1-positive endolysosomes (confocal laser scanning microscopy (CLSM), Figure 2(A) and quantifications with LysoQuant^[26]^, a deep-learning-based analysis software for segmentation and classification of fluorescence images, Figure 2(E))^[10]^. Lysosomal clearance of ATZ requires the contact between ER-derived structures containing ATZ polymers (ER in Figure 1(C), highlighted in red in the immunoelectron microscopy (IEM) micrograph in Figure 2B) and the membrane of degradative endolysosomes (EL in Figure 1(C), highlighted in green in Figure 2(B)). The contact between ER and endolysosome is followed by a membrane fusion event involving the SNARE proteins Syntaxin17 (STX17) and VAMP8 that we have previously characterized^[10]^, and that delivers the ATZ polymers from the ER lumen into the lumen of the RAB7/LAMP1-positive degradative endolysosomes (the arrow in Figure 1(C) and the blue asterisk in Figure 2(B) show the opening between the ER and the EL membranes). In cells lacking STX17 generated by CRISPR/Cas9 genome editing^[10]^, the delivery of ATZ within the LAMP1-positive degradative compartments is strongly inhibited (Figure 2(C,E))^[10]^. The ER structures segregating ATZ polymers (Figure 2(D), highlighted in red) dock at the limiting membrane of the RAB7/LAMP1-positive endolysosomes (highlighted in green) but fail to fuse and to deliver ATZ polymers in cells with dysfunctional SNARE complexes (Figure 2(C–E)).

NHK, a folding-defective variant of alpha1-antitrypsin that is mainly degraded by the ubiquitin/proteasome system via ERAD^[12,23–25]^ is only poorly delivered within the endolysosomal compartment in MEF (Figure 2(E,F)) and deletion of STX17 does not affect its intracellular distribution (Figure 2(E,G))^[12]^.

### Luminal accumulation of ATZ promotes formation of ER-endolysosomes contact sites

Next, we checked by CLSM whether the expression of the ERLAD client ATZ, or of the ERAD client NHK affects the proximity between the ER and the endolysosomes. The experiments were performed in CRISPR STX17 cells, where the absence of the fusion machinery that drives the transfer of ATZ polymers from the ER to the lumen of endolysosomes eventually stabilizes the juxtaposition of the two organelles^[10]^. The Mander’s correlation coefficient used to establish the level of co-localization between the ER marker protein VAPA and the endolysosomal marker protein LAMP1^[27–29]^ reveals higher values in cells expressing ATZ than in cells mock-transfected or expressing the folding-defective ERAD client NHK (Figure 2(H–K), respectively). Thus, the expression of a misfolded polypeptide that must be cleared by the endolysosomal system enhances the contacts between the biosynthetic ER compartment and the degradative endolysosomal compartments, whereas the expression of a misfolded protein mainly cleared by the ubiquitin/proteasome system does not.

### The intraluminal expression of ATZ enhances the co-localization of contact sites regulators at the membrane of endolysosomes

**Figure 3.**
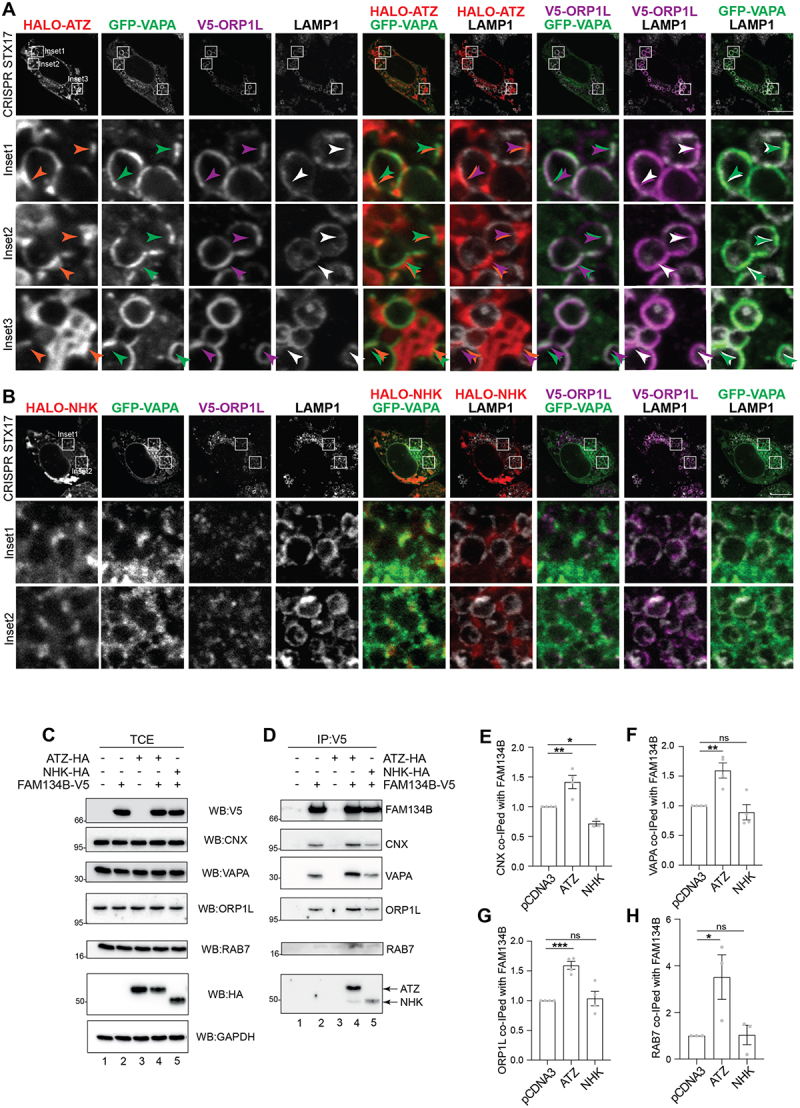
Expression of ATZ induces the formation of CNX:FAM134B:VAPA:ORP1L:RAB7 complexes (A) CLSM analysis showing GFP-VAPA, V5-ORP1L and LAMP1 distribution in HALO-ATZ transfected CRISPR STX17 MEFs treated with 50 nM BafA1. (B) same as a for cells expressing HALO-NHK. (C) WB of the total cell extract (TCE) of HEK293 cells transfected with empty vector (lane 1), FAM134B‐V5 (lane 2), ATZ‐HA (lane 3), FAM134B‐V5 and ATZ‐HA (lane 4), or FAM134B‐V5 and NHK‐ HA (lane 5), incubated for 6 h with 100 nM BafA1 and then lysed with 2% CHAPS. Membranes were probed with anti‐V5, anti‐CNX, anti VAPA, anti‐ORP1L, anti-Rab7, anti‐HA and anti-GAPDH antibodies, respectively (D) WB of the anti‐V5 immunocomplexes of C. Membranes were probed with anti‐V5, anti‐CNX, anti-VAPA, anti‐ORP1L, anti-Rab7 and anti‐HA antibodies, respectively. (E) quantification of the co-immunoprecipitation of CNX with FAM134B observed in D, the association in mock-transfected cells is set to 1. One‐way ANOVA and Dunnett’s multiple comparisons test, *P < 0.1, **P < 0.01. F = 23.06. *n* = 4 independent experiments for pCDNA3 and ATZ, *n* = 3 independent experiments for NHK. (F) quantification of the co-immunoprecipitation of VAPA with FAM134B observed in D. One‐way ANOVA and Dunnett’s multiple comparisons test, ^ns^*P* > 0.05, **P < 0.01. F = 13.00. *n* = 4 independent experiments (G) quantification of the co-immunoprecipitation of ORP1L with FAM134B observed in D. One‐way ANOVA and Dunnett’s multiple comparisons test, ^ns^*P* > 0.05, ***P < 0.001. F = 26.58. *n* = 4 independent experiments (H) quantification of the co-immunoprecipitation of RAB7 with FAM134B observed in D. One‐way ANOVA and Dunnett’s multiple comparisons test, ^ns^*P* > 0.05, *P < 0.1. *n* = 3 independent experiments F = 5.540. Scale bar: 10 µm.

To confirm that the luminal accumulation of the ERLAD client ATZ promotes the formation of contacts between ER subdomains and endolysosomes, we monitored the subcellular localization of proteins reportedly involved in ER-endolysosomes contact sites, which are set by the VAPA localized at the ER membrane, the small GTPase RAB7 associated at the membrane of LAMP1-positive endolysosomes, and the cytoplasmic protein ORP1L that bridges the two^[30–33]^. Analyses by CLSM in CRISPR STX17 cells reveal that in cells expressing HALO-ATZ, the misfolded polypeptide (orange arrowheads in the Insets, Figure 3(A)) co-localizes with GFP-VAPA (green arrowheads) and with V5-ORP1L (purple arrowheads) at the limiting membranes of LAMP1-positive endolysosomes (white arrowheads). To note, the ATZ-enriched regions in contact with LAMP1 positive endolysosomes are somewhat wider in cells expressing high levels of VAPA and ORP1L, where ER-endolysosomes MCS are more frequent (Figure 3(A)) than in cells mock-transfected (Figure 2(C)). In cells expressing the ERAD client NHK, we observe two major differences that suggest that the ERAD client does not induce VAPA/ORPL1 contact sites formation. First, the lack of condensation of the V5-ORP1L signal in correspondence to the LAMP1 signal at the limiting membrane of the endolysosomal compartments (Figure 3(B), compare the subcellular distribution of V5-ORP1L in the Inset, with the corresponding Inset of Figure 3(A)).

Second, and consistent with the role of ORP1L in bridging the ER-localized VAPA protein by anchoring RAB7 GTPase located at the endolysosomal membrane^[30–33]^, GFP-VAPA does not accumulate near the rim of LAMP1 enriched endolysosomes (Figure 3(B)), as a significant difference with cells expressing the ERLAD client ATZ (Figure 3(A)).

### The intraluminal expression of ATZ promotes the formation of CNX:FAM134B:VAPA:ORP1L:RAB7 complexes

Few considerations led us to examine a possible induction of protein complexes engaging VAPA, ORP1L, and RAB7 that control ER-endolysosomes contact sites in cells accumulating ATZ. First, the increased interaction between proteins involved in the generation/stabilization of ER-endolysosomes contact sites seems a *conditio sine qua non* to explain the imaging data shown in (Figures 2 and 3(A,B)) testifying the spatial co-localization of these proteins. Second, previous experiments from our lab showed that the luminal accumulation of ATZ relies on the persistent association of the misfolded polypeptide with the ER-resident lectin chaperone CNX via the N-linked glycan at position 83 of ATZ. The long-lasting association promotes the engagement of the ER-phagy receptor FAM134B^[9,10]^. Third, VAPA is a major interactor of the ER-phagy receptor FAM134B^[22]^. However, the function of the VAPA:FAM134B complexes is unknown. To verify if luminal accumulation of misfolded proteins affects the propensity of ER and endolysosomes to engage in contacts, HEK293 cells were mock-transfected (Figure 3C, lane 1), or were transfected with plasmids for expression of FAM134B-V5 (lane 2), ATZ-HA (lane 3), FAM134B-V5 and ATZ-HA (lane 4), or FAM134B-V5 and NHK-HA (lane 5). FAM134B was immunoisolated with an anti-V5 antibody from lysates of cells solubilized with the zwitterionic detergent CHAPS to preserve the association of the ER-phagy receptor with ectopically expressed and endogenous proteins in functional complexes^[9,10]^ (Figure 3(D)). In mock-transfected cells, no V5 signal is visible, confirming the specificity of the anti-V5 antibody (Figure 3(D), lane 1). Immunoisolation of ectopically expressed FAM134B confirms the constitutive association of endogenous CNX (lane 2)^[10]^ and of endogenous VAPA (lane 2)^[22]^ revealed with the corresponding antibodies in the western blots. Significantly, these complexes also contain endogenous ORP1L and low, but detectable amounts of endogenous RAB7 (lane 2), reporting on the constitutive presence of ER-endolysosomes contact sites^[30,32]^. The luminal accumulation of the ERLAD client ATZ enhances the formation/stability of the functional complexes between FAM134B and endogenous CNX (Figure 3(D), lanes 2 *vs*. 4, 3E), as we previously reported^[10]^. Our new tests add that ATZ expression also substantially increases the association of endogenous VAPA, ORP1L, and RAB7 (lanes 2 *vs*. 4, 3F-3 H) with the functional FAM134B:CNX complex that ensures lysosomal clearance of ATZ.

NHK, a misfolded polypeptide cleared from cells via ERAD^[12,23–25]^, only weakly engages FAM134B and the ERLAD machinery (lane 5)^[12]^ and its luminal expression does not affect the co-precipitation of endogenous CNX, VAPA, ORP1L, and RAB7 with FAM134B (Figure 3(D), lanes 2 *vs*. 5 and 3E-3 H).

### VAPA is required for ERLAD of ATZ

**Figure 4.**
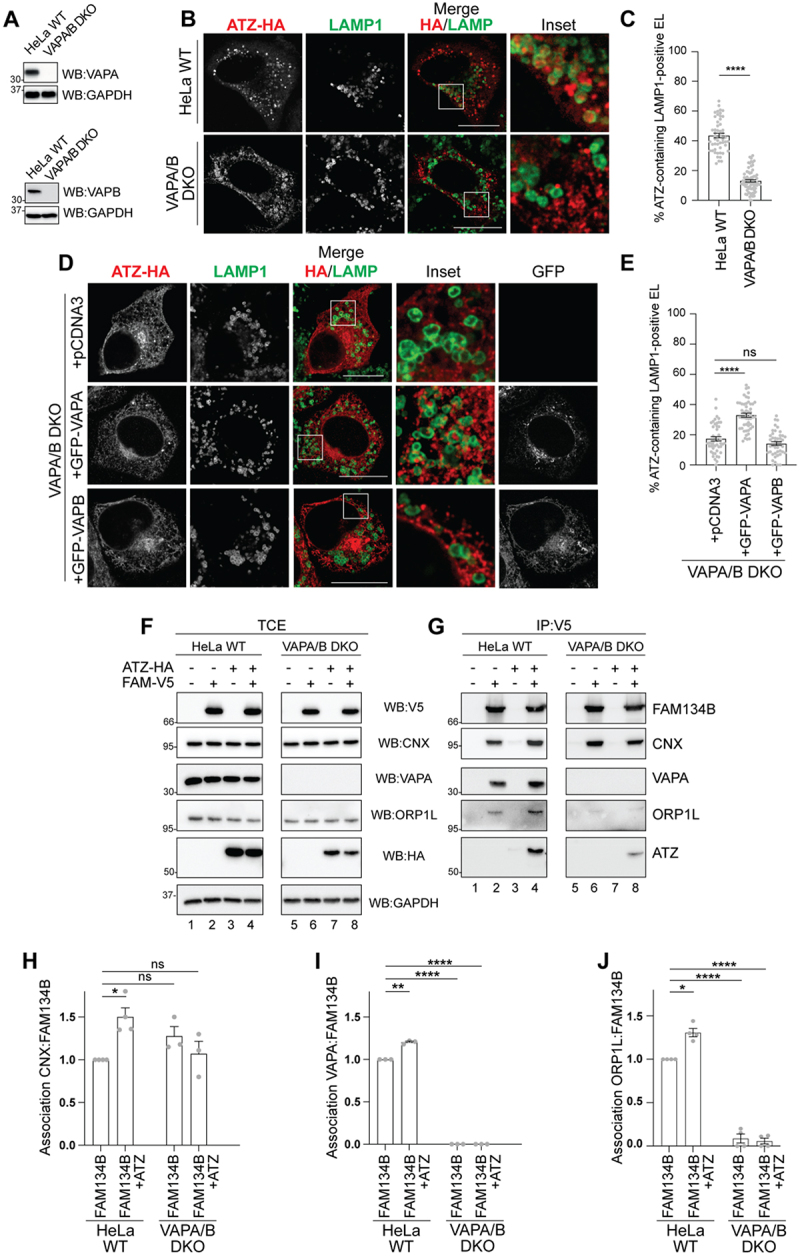
VAPA is required for ATZ delivery to endolysosomes (A) WB analysis showing VAPA (upper) and VAPB (lower panels) down-regulation efficiency for HeLa VAPA/B DKO cells. (B) CLSM analysis of ATZ-HA delivery to LAMP1-positive endolysosomes in HeLa WT (upper panels) and in HeLa VAPA/B DKO cells (lower panels) treated with 100 nM BafA1. (C) LysoQuant quantification of B (*n* = 56 and 71 cells of three independent experiments, respectively). Unpaired two‐tailed *t*‐ test, ^ns^*P* > 0.05, ****p < 0.0001. *t* = 16.88. (D) CLSM analysis of ATZ-HA delivery to LAMP1-positive endolysosomes in HeLa VAPA/B DKO cells treated with 100 nM BafA1 mock transfected (upper panels), transfected with GFP-VAPA (middle panels) or with GFP-VAPB (lower panels). (E) quantification of D (*n* = 46, 51, and 43 cells of three independent experiments, respectively). One‐way ANOVA and Dunnett’s multiple comparisons test, **P < 0.01, ****P < 0.0001. F = 51.21. (F) WB of the total cell extract (TCE) of HeLa WT cells (lanes 1–4) and HeLa VAPA/B DKO cells (lanes 5–8) transfected with empty vector (lanes 1 and 5), FAM134B‐V5 (lanes 2 and 6), ATZ‐HA (lanes 3 and 7), or FAM134B‐V5 and ATZ‐HA (lanes 4 and 8), incubated for 6 h with 100 nM BafA1 and then lysed with 2% CHAPS. Membranes were probed with anti‐V5, anti‐CNX, anti-VAPA, anti‐ORP1L, anti‐HA and anti-GAPDH antibodies, respectively. (G) WB of anti‐V5 immunocomplexes of F. Membranes were probed with anti‐V5, anti‐CNX, anti-VAPA, anti‐ORP1L, and anti‐HA antibodies, respectively. H) quantification of the association CNX:FAM134B observed in G. One‐way ANOVA and Dunnett’s multiple comparisons test, ^ns^*P* > 0.05, *P < 0.1. F = 11.65. *n* = 4 independent experiments for HeLa WT and *n* = 3 for HeLa VAPA/B DKO cells. (I) quantification of the association VAPA:FAM134B observed in G. One‐way ANOVA and Dunnett’s multiple comparisons test, **P < 0.01, **** *P* < 0.0001. F = 329.2. *n* = 3 independent experiments (J) quantification of the association ORP1L:FAM134B observed in G. One‐way ANOVA and Dunnett’s multiple comparisons test, *P < 0.1, ****P < 0.0001. F = 148.9. *n* = 4 independent experiments. Scale bar: 10 µm.

VAP proteins are present in several flavors in the mammalian ER, VAPA, and VAPB being involved in ER-endolysosomes, and ER-mitochondria contact sites, respectively^[31,34,35]^. To assess the involvement of the VAPA:ORP1L complex in the clearance of misfolded ATZ from the ER, we first monitored by CLSM the lysosomal delivery of ATZ in wild-type HeLa cells and in HeLa cells, where VAPA and VAPB have been deleted (Figure 4(A))^[36]^. The deletion of VAPA/B (Figure 4(B), lower panels and quantification by LysoQuant, 4(C)) substantially inhibits delivery of ATZ within LAMP1-positive endolysosomes compared to wild-type HeLa cells (Figure 4(B), upper panels and 4(C)). The back-transfection of GFP-VAPA partially restores the lysosomal delivery of ATZ in VAPA/B double KO cells (Figure 4(D), middle panel, 4(E)). The back-transfection of GFP-VAPB does not restore it (Figure 4(D), lower panel, 4(E)). The statistically significant, yet incomplete recovery of ATZ delivery within the LAMP1-positive degradative compartment upon GFP-VAPA back-transfection can possibly be ascribed to sub-optimal participation of ectopically expressed VAPA in functional MCS complexes. These data show that VAPA, and not VAPB, plays a crucial role in the stabilization of ER-endolysosome contact sites that promote ATZ clearance from the ER. Co-immunoprecipitation assays show that in cells lacking VAPA (Figure 4(F,G) lanes 5–8) the luminal accumulation of ATZ does not augment the interaction between (i.e., the co-precipitation of) CNX and FAM134B (Figure 4(G), lanes 5–8 and quantification in 4 (H)). The absence of VAPA abolishes the engagement of endogenous ORP1L in the complex that should warrant the formation of the ER-endolysosome contact sites (Figure 4(G), compare lane 4 (co-precipitation of the contact sites components in wild-type HeLa cells) *vs*. lane 8 (the same in HeLa cells lacking the VAPA component), quantifications in 4(I-J)). This confirms, in another cell line, that FAM134B associates with VAPA (as shown in Figure 3 for MEF)^[22,37–42]^ and that VAPA is required to engage ORP1L in functional complexes promoting the formation of contacts between the biosynthetic and the degradative compartments.

### ORP1L and its association with VAPA are required for ERLAD of ATZ

**Figure 5.**
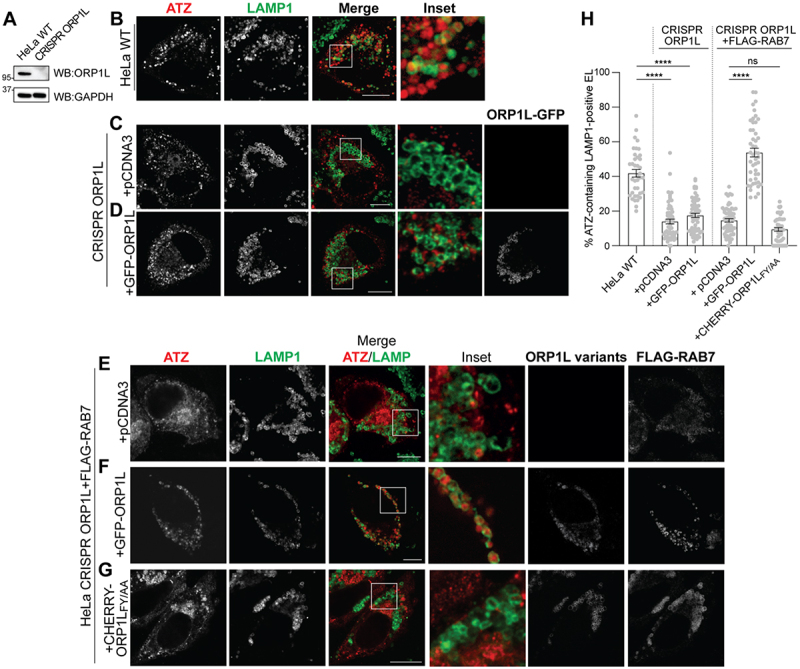
ORP1L is required for ATZ delivery to endolysosomes (A) WB analysis showing the knockout of ORP1L in HeLa cells. (B) CLSM analysis of ATZ-HA delivery to LAMP1-positive endolysosomes in HeLa WT cells treated with 100 nM BafA1. (C) CLSM analysis of ATZ-HA delivery to LAMP1-positive endolysosomes in HeLa CRISPR ORP1L cells mock transfected, treated with 100 nM BafA1. (D) CLSM analysis of ATZ-HA delivery to LAMP1-positive endolysosomes in HeLa CRISPR ORP1L cells transfected with GFP-ORP1L, treated with 100 nM BafA1. (E) CLSM analysis of HALO-ATZ delivery to LAMP1-positive endolysosomes in HeLa CRISPR ORP1L cells transfected with FLAG-Rab7, treated with 100 nM BafA1. (F) CLSM analysis of HALO-ATZ delivery to LAMP1-positive endolysosomes in HeLa CRISPR ORP1L cells transfected with GFP-ORP1L and FLAG-Rab7, treated with 100 nM BafA1. (G) CLSM analysis of ATZ-HA delivery to LAMP1-positive endolysosomes in HeLa CRISPR ORP1L cells transfected with CHERRY-ORP1L_FY/AA_ and FLAG-Rab7, treated with 100 nM BafA1. (H) quantification of B-G (*n* = 38, 57, 64, 59, 47, and 48 cells of three independent experiments, respectively). One‐way ANOVA and Dunnett’s multiple comparisons test, ^ns^*P* > 0.05, **P < 0.01, ****P < 0.0001. F = 124.3. Scale bar: 10 µm.

Deletion of ORP1L by CRISPR/Cas9 genome editing (Figure 5(A)) substantially impairs lysosomal delivery of ATZ polymers in HeLa cells (Figure 5(B,C) and quantifications in 5 H, +pCDNA3). The lysosomal delivery of ATZ is only poorly, if at all, restored upon back transfection of ORP1L alone (Figure 5(D,H), +ORP1L-GFP). However, when ORP1L is co-expressed with RAB7, the ORP1L interactor at the membrane of endolysosomes^[30]^, ATZ delivery is effectively restored (Figure 5(F,H)).

**Figure 6.**
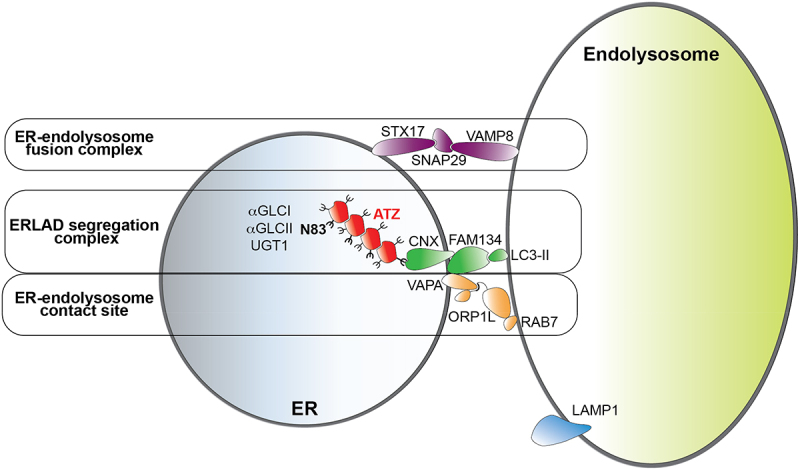
Functional complexes involved in ERLAD of ATZ polymers. Luminal expression of ATZ polymers and persistent cycles of de-glucosylation by α-glucosidase I and II (αGLCI, αGLCII) and re-glucosylation by UDP-glucose:glycoprotein glucosyltransferase 1 (UGT1) of the oligosaccharide at position 83 of the ATZ polypeptide prolong the association of ATZ polymers with the lectin chaperone calnexin (CNX)^[9]^. This leads to the formation of a segregation complex between CNX and the LC3-II interacting ER-phagy receptor FAM134B^[10]^and, as shown here, stabilizes ER-endolysosome contacts involving the FAM134B interactor VAPA, the endolysosome-associated small GTPase RAB7 and the connecting protein ORP1L. Membrane fusion events controlled by the SNARE proteins STX17, SNAP29 and VAMP8 allow delivery of ATZ polymers within endolysosomes for clearance^[10]^.

The co-transfection of ORP1L_FY-AA_, a mutant form of ORP1L that does not bind VAPA^[43]^, with RAB7 does not rescue ATZ delivery to the degradative compartments (Figure 5(G,H)). All in all, these data confirm that multiprotein complexes involved in VAPA:ORP1L:RAB7 contact sites contribute with the CNX:FAM134B segregation complex and the STX17:SNAP29:VAMP8 membrane fusion complex to the efficient clearance from the ER of ATZ polymers via LC3-dependent delivery to LAMP1-positive endolysosomal compartments (Figure 6).

## Discussion

The ER is site of protein synthesis and maturation. Sophisticated quality control pathways ensure that native and functional polypeptides are transported at their *intra-* or *extra*-cellular site of activity^[44]^. Terminally misfolded polypeptides are removed from the ER via proteasomal (ERAD), or lysosomal (ERLAD) pathways^[1–8]^. The mechanistic dissection of ERLAD is still in its infancy, and the study of the client specific pathways that deliver misfolded proteins from their site of generation (the ER) to the endolysosomal (or vacuolar in yeast and plants) degradative compartments is shedding light on intracellular quality control, client segregation, ER remodeling, transport, and clearance pathways^[4]^. As thoroughly reviewed elsewhere^[4]^, the autophagic and non-autophagic routes of misfolded proteins for lysosomal degradation include a detour via the plasma membrane (e.g., for GPI anchored misfolded polypeptides^[45–47]^), the capture of ER portions or of ER exit sites containing misfolded proteins by double membrane autophagosomes or by endolysosomes (e.g., for mutant forms of pro-collagen^[9,48,49]^), the fusion of ER portions containing misfolded proteins with degradative endolysosomes (e.g., for ATZ polymers^[9,10,12]^). This latter type of ERLAD, the LC3-dependent lysosomal clearance of misfolded polypeptides, relies on the persistent re-glucosylation of the misfolded polypeptide that prolongs engagement of the lectin chaperone CNX with subsequent segregation in ER subdomains displaying the ER-phagy receptor FAM134B at the limiting membrane. These ER subdomains eventually deliver their toxic content for clearance within RAB7/LAMP1-positive endolysosomes upon SNARE proteins-driven fusion^[9,10,12]^. Notably, genetic variants of FAM134 proteins are putative disease modifiers in children expressing ATZ^[50]^, revealing that this catabolic pathway, if defective, may significantly worsen disease progression and hepatic toxicity linked to the Z mutation, eventually leading to the necessity for a liver transplant. The mechanistic dissection of this pathway led to the characterization of a constitutively present multimeric protein complex composed of CNX:FAM134B:VAPA:ORP1L:RAB7, whose abundance/stability is enhanced in cells expressing ATZ polymers. It is likely that the constitutive levels of the multiprotein complex identified in this study warrant the maintenance of ER homeostasis by ensuring the efficient removal of by-products of protein biosynthesis that are continuously generated in the ER lumen. In this complex, the ER resident tail-anchored protein VAPA^[14–33]^, a major interactor of the ER-phagy receptor FAM134B at the ER membrane with unknown function in ER-phagy so far^[22]^ and the endolysosome-associated small GTPase RAB7 are bridged by the cytoplasmic ORP1L protein to form ER:endolysosome MCS that are required for efficient clearance of ATZ polymers from the ER. It is conceivable that ERLAD of other clients that proceeds via distinct mechanistic pathways such as macro-ER-phagy or the RESET pathway (misfolded pro-collagen^[9,48]^ or glycosylphosphatidylinositol-anchored proteins^[46]^) does not require the stabilization of contact sites between the ER and the endolysosomes. MCS between ER and endolysosomes are relevant for the ORP1L-driven transfer of lipids from the site of their biosynthesis (the ER) to endolysosomal membranes, control endolysosome maturation and, according to the results of our research, promote delivery of toxic macromolecules, in this case, disease-causing misfolded polypeptides, for removal from our cells. Notably, VAPA is a major partner of a series of ER-phagy receptors in yeast and mammalian cells^[22,37,38,40,51]^. For soluble ER-phagy receptors (CALCOCO1 in mammalian cells^[38]^ and Epr1 in yeast^[40]^), association with the ER proteins of the VAP family recruits the receptor at the ER membrane upon activation of the ER-phagy program. Our data imply that these associations could serve to recruit the receptor at ER subdomains in close contact with endolysosomal compartments. Significantly, VAPA has recently been identified as a major client of ER-phagy^[39,41]^. If VAPA and/or ER-endolysosomes contact sites established by VAPA contribute to other ER-phagy programs in addition to ERLAD, remains to be established.

## Material and methods

### Expression plasmids

pCDNA3.1 ATZ-HA, pCDNA3.1 NHK-HA, pCDNA3.1 FAM134B-V5, pCDNA3.1 HALO-ATZ, and pCDNA3.1 HALO-NHK were prepared as previously described^[10,52–55]^. GFP-ORP1L was a kind gift of J. Neefjes. CHERRY-ORP1L_FY/AA_, pEF FLAG-RAB7, GFP-VAPA, and GFP-VAPB were kind gifts of N. Ridgway, F. Reggiori, and P. De Camilli. pCDNA5 V5-ORP1L was generated by exchanging GFP of GFP-ORP1L with V5tag.

### Antibodies

All the antibodies used in the study were listed in Table S1. Commercial antibodies used in the study were rabbit HA (Sigma), rat HA (Novus), V5 (Thermo Fisher), GAPDH (Thermo Fisher), VAPA (Bioss), VAPB (Abcam), ORP1L (Abcam), RAB7 (Abcam), FLAG (Sigma), LAMP1 clone 1D4B (Hybridoma Bank, 1D4B was deposited to the DSHB by J.T. August), LAMP1 clone H3A4 (Hybridoma Bank, 1D4B was deposited to the DSHB by August, J.T). CNX was gift A. Helenius. Alexa-conjugated and HRP-conjugated secondary antibodies were from Thermo Fisher Scientific and Jackson ImmunoResearch, respectively.

### Cell culture, transient transfection, and inhibitors

HEK293 cells were purchased from ATCC. STX17-deleted mouse embryonic fibroblasts (MEFs) were generated by CRISPR/Cas9 genome editing as previously described^[10]^. HeLa WT and VAPA/B DKO cells were a kind gift of P. De Camilli; HeLa CRISPR mock and CRISPR ORP1L cells were a kind gift of N. Ridgway. All cell lines were grown in DMEM supplemented with 10% FBS at 37°C, 5% CO_2_. Cells were transfected with JetPrime (Polyplus) following manufacturer’s instructions. Bafilomycin A1 (Sigma) was used 100 mM for 12 h or 50 mM for 6 h if not elsewhere stated.

### Cell lysis, immunoprecipitation, and Western blot

Cells were plated on Poly-Lys (Sigma) and treated as indicated. Cells were then washed with ice cold PBS with 20 mM NEM (Sigma) and lysed with 2% CHAPS (Anatrace) in HEPES buffer pH 6.8 supplemented with protease inhibitors for 20 minutes on ice.

Post-nuclear supernatants (PNS) were isolated by centrifugation at 10,600 g for 10 minutes. Samples were then denatured and reduced with 2% SDS- and 100 mM DTT-containing sample buffer at 95°C for 5 min.

For immunoprecipitation assays, CHAPS lysates were incubated with V5-agarose beads (Sigma) at 4°C overnight. After two washes with 0.5% CHAPS, immunocomplexes were denatured and reduced with 6% SDS- and 10 mM DTT-containing sample buffer at 95°C for 5 minutes.

Samples were then loaded on 10% Tris-glycine SDS-PAGE gels and transferred to PVDF membranes using the Trans-Blot Turbo Transfer System (Bio-Rad). Membranes were blocked with 10% Milk in TBS-T and incubated with primary and then HRP-conjugated secondary antibodies diluted in TBS-T. Membranes were then developed with WesternBright ECL or Quantum (Advansta) and signals detected with FusionFX7 VILBER (Witec). Quantifications were performed with Fiji/ImageJ.

### Confocal laser scanning microscopy

Cells were plated on Alcian blue (Sigma)-treated glasses and then transfected, treated with BafA1, and subsequently fixed with 3.7% formaldehyde in PBS at room temperature for 15 minutes. HALO-tagged proteins were labeled with TMR HaLo ligand (Promega) for 12 h prior fixation.

Fixed cells were permeabilized for 15 minutes with permeabilization solution (PS, 0.05% saponin, 10% goat serum, 10 mM HEPES, 15 mM glycine). Primary antibody was diluted 1:100 with PS solution and incubated for 90 minutes. After washing for three times for 5 minutes, cells were then stained with Alexa-conjugated secondary antibodies for 45 minutes. Cells were rinsed with PS for three times and water and mounted with mount liquid anti-fade (Abberior).

Confocal images were acquired with Leica TCS SP5 or Leica Stellaris SP8 microscope with a Leica HCX PL APO lambda blue 63.0 × 1.40 OIL UV objective.

The quantifications of EL delivery per cell were executed with LysoQuant, an unbiased and automated deep learning tool for fluorescent image quantification^[26]^. Image processing was performed with Fiji/ImageJ and Adobe Photoshop.

### Immunogold electron microscopy

Cells were plated on alcian blue coverslips, transfected and fixed with 3.7% paraformaldehyde (PFA, Sigma). After washes in PBS and 50 mM glycine, cells were permeabilized with 0.25% saponin, 0.1% BSA, and blocked in blocking buffer (0.2% BSA, 5% goat serum, 50 mM NH_4_Cl, 0.1% saponin, 20 mM PO_4_ buffer, 150 mM NaCl). Staining with primary antibodies and nanogold‐labeled secondary antibodies (Nanoprobes) was performed in blocking buffer at room temperature. Cells were re‐fixed in 1% glutaraldehyde, and nanogold was enlarged with gold enhancement solution (Nanoprobes) according to the manufacturer’s instructions. Cells were post‐fixed with osmium tetroxide, embedded in Epon, and processed into ultrathin slices. After contrasting with uranyl acetate and lead citrate, sections were analyzed with a Zeiss LEO 512 electron microscope. Images were acquired by a 2k bottom‐mounted slow‐scan Proscan camera controlled by EsivisionPro 3.2 software. Image analysis and quantification were performed with FIJI.

### Statistical analysis

Plots and statical analyses were performed with GraphPad Prism 9 (GraphPad Software Inc.). To assess the statistical significance, one-way ANOVA with Dunnett’s multiple comparisons test and unpaired two-tailed *t-*test were performed. *P*-value <0.05 (for one-way ANOVA with Dunnett’s multiple comparisons test) and *P*-value <0.05 (for *t*-test) were considered statistically significant.

## Data Availability

The authors confirm that the data supporting the findings of this study are available within the article and its supplementary materials.

## References

[1] Christianson JC, Jarosch E, Sommer T. Mechanisms of substrate processing during ER-associated protein degradation. Nat Rev Mol Cell Biol. 2023;24(11):777–15. doi: 10.1038/s41580-023-00633-8 37528230

[2] Ferro-Novick S, Reggiori F, Brodsky JL. Er-phagy, ER homeostasis, and ER quality control: implications for disease. Trends Biochem Sci. 2021;46(8):630–639. doi: 10.1016/j.tibs.2020.12.013 33509650 PMC8286283

[3] Sun Z, Brodsky JL. Protein quality control in the secretory pathway. J Cell Biol. 2019;218(10):3171–3187. doi: 10.1083/jcb.201906047 31537714 PMC6781448

[4] Rudinskiy M, Molinari M. Er-to-lysosome-associated degradation in a nutshell: mammalian, yeast, and plant ER-phagy as induced by misfolded proteins. FEBS Lett. 2023;597(15):1928–1945. doi: 10.1002/1873-3468.14674 37259628

[5] Wu SA, Li ZJ, Qi L. Endoplasmic reticulum (ER) protein degradation by ER-associated degradation and ER-phagy. Trends Cell Biol. 2025;35(7):576–591. doi: 10.1016/j.tcb.2025.01.002 39909774 PMC12227305

[6] Knupp J, Pletan ML, Arvan P, et al. Autophagy of the ER: the secretome finds the lysosome. Febs J. 2023;290(24):5656–5673. doi: 10.1111/febs.16986 37920925 PMC11044768

[7] Salomo-Coll C, Jimenez-Moreno N, Wilkinson S. Lysosomal degradation of ER client proteins by ER-phagy and related pathways. J Mol Biol. 2025;437(18):169035. doi: 10.1016/j.jmb.2025.169035 39993592

[8] Fregno I, Molinari M. Proteasomal and lysosomal clearance of faulty secretory proteins: ER-associated degradation (ERAD) and ER-to-lysosome-associated degradation (ERLAD) pathways. Crit Rev Biochem Mol Biol. 2019;54(2):153–163. doi: 10.1080/10409238.2019.1610351 31084437

[9] Fregno I, Fasana E, Solda T, et al. N-glycan processing selects ERAD-resistant misfolded proteins for ER-to-lysosome-associated degradation. Embo J. 2021;40(15):e107240. doi: 10.15252/embj.2020107240 34152647 PMC8327951

[10] Fregno I, Fasana E, Bergmann TJ, et al. Er-to-lysosome-associated degradation of proteasome-resistant ATZ polymers occurs via receptor-mediated vesicular transport. Embo J. 2018;37(17):e99259. doi: 10.15252/embj.201899259 30076131 PMC6120659

[11] De Leonibus C, Maddaluno M, Ferriero R, et al. Sestrin2 drives ER-phagy in response to protein misfolding. Dev Cell. 2024;59(16):2035–2052 e10. doi: 10.1016/j.devcel.2024.07.004 39094564 PMC11338521

[12] Fasana E, Fregno I, Galli C, et al. Er-to-lysosome-associated degradation acts as failsafe mechanism upon ERAD dysfunction. EMBO Rep. 2024;25(6):2773–2785. doi: 10.1038/s44319-024-00165-y 38773321 PMC11169228

[13] Marciniak SJ, Ordonez A, Dickens JA, et al. New concepts in alpha-1 antitrypsin deficiency disease mechanisms. Ann Am Thorac Soc. 2016;13(4):289–296. 10.1513/AnnalsATS.201506-358KV 27564663

[14] Voeltz GK, Sawyer EM, Hajnóczky G, et al. Making the connection: how membrane contact sites have changed our view of organelle biology. Cell. 2024;187(2):257–270. doi: 10.1016/j.cell.2023.11.040 38242082 PMC11830234

[15] Calì T, Bayer EM, Eden ER, et al. Key challenges and recommendations for defining organelle membrane contact sites. Nat Rev Mol Cell Biol. 2025;26(10):1–21. doi: 10.1038/s41580-025-00864-x 40550870

[16] Friedman JR, Dibenedetto JR, West M, et al. Endoplasmic reticulum-endosome contact increases as endosomes traffic and mature. Mol Biol Cell. 2013;24(7):1030–1040. doi: 10.1091/mbc.e12-10-0733 23389631 PMC3608491

[17] Huotari J, Helenius A. Endosome maturation. Embo J. 2011;30(17):3481–3500. doi: 10.1038/emboj.2011.286 21878991 PMC3181477

[18] Kilpatrick BS, Eden ER, Hockey LN, et al. An endosomal NAADP-sensitive two-pore Ca(2+) channel regulates ER-endosome membrane contact sites to control growth factor signaling. Cell Rep. 2017;18(7):1636–1645. doi: 10.1016/j.celrep.2017.01.052 28199837 PMC5318655

[19] Wenzel EM, Raiborg C. Er-endosome contacts master the ins and outs of secretory endosomes. J Cell Biol. 2022;221(12):e202210033. doi: 10.1083/jcb.202210033 36355088 PMC9652769

[20] Olkkonen VM, Ikonen E. Getting to grips with the oxysterol-binding protein family - a forty year perspective. Contact (Thousand Oaks). 2024;7:1–12. doi: 10.1177/25152564241273598 PMC11359446 39210909

[21] Cremer T, Neefjes J, Berlin I. The journey of Ca(2+) through the cell - pulsing through the network of ER membrane contact sites. J Cell Sci. 2020;133(24):1–12. doi: 10.1242/jcs.249136 33376155

[22] Rudinskiy M, Galli C, Raimondi A, et al. The intrinsically disordered regions of organellophagy receptors are interchangeable and control organelle fragmentation. Nat Cell Biol. 2025;27(9):1431–1447. doi: 10.1038/s41556-025-01728-4 40760246 PMC12431859

[23] Sifers RN, Brashears-Macatee S, Kidd VJ, et al. A frameshift mutation results in a truncated alpha 1-antitrypsin that is retained within the rough endoplasmic reticulum. J Biol Chem. 1988;263(15):7330–7335. doi: 10.1016/S0021-9258(18)68646-6 3259232

[24] Liu Y, Choudhury P, Cabral CM, et al. Oligosaccharide modification in the early secretory pathway directs the selection of a misfolded glycoprotein for degradation by the proteasome. J Biol Chem. 1999;274(9):5861–5867. doi: 10.1074/jbc.274.9.5861 10026209

[25] Liu Y, Choudhury P, Cabral CM, et al. Intracellular disposal of incompletely folded human alpha1-antitrypsin involves release from calnexin and post-translational trimming of asparagine-linked oligosaccharides. J Biol Chem. 1997;272(12):7946–7951. doi: 10.1074/jbc.272.12.7946 9065464

[26] Morone D, Marazza A, Bergmann TJ, et al. Deep learning approach for quantification of organelles and misfolded polypeptide delivery within degradative compartments. Mol Biol Cell. 2020;31(14):1512–1524. doi: 10.1091/mbc.E20-04-0269 32401604 PMC7359569

[27] Manders EMM, Verbeek FJ, Aten JA. Measurement of co-localization of objects in dual-colour confocal images. J Microsc. 1993;169(3):375–382. doi: 10.1111/j.1365-2818.1993.tb03313.x 33930978

[28] Nguyen TT, Voeltz GK. An ER phospholipid hydrolase drives ER-associated mitochondrial constriction for fission and fusion. Elife. 2022;11:e84279. doi: 10.7554/eLife.84279 36448541 PMC9725753

[29] Wijdeven RH, Janssen H, Nahidiazar L, et al. Cholesterol and ORP1L-mediated ER contact sites control autophagosome transport and fusion with the endocytic pathway. Nat Commun. 2016;7(1):1–14. doi: 10.1038/ncomms11808 PMC4906411 27283760

[30] Rocha N, Kuijl C, van der Kant R, et al. Cholesterol sensor ORP1L contacts the ER protein VAP to control Rab7-RILP-p150 glued and late endosome positioning. J Cell Biol. 2009;185(7):1209–1225. doi: 10.1083/jcb.200811005 19564404 PMC2712958

[31] Murphy SE, Levine TP. VAP, a versatile access point for the endoplasmic reticulum: review and analysis of FFAT-like motifs in the VAPome. Biochim Et Biophys Acta-Mol Cell Biol Lipids. 2016;1861(8):952–961. doi: 10.1016/j.bbalip.2016.02.009 26898182

[32] Johansson M, Lehto M, Tanhuanpaa K, et al. The oxysterol-binding protein homologue ORP1L interacts with Rab7 and alters functional properties of late endocytic compartments. Mol Biol Cell. 2005;16(12):5480–5492. doi: 10.1091/mbc.e05-03-0189 16176980 PMC1289395

[33] Neefjes J, Cabukusta B. What the VAP: the expanded VAP family of proteins interacting with FFAT and FFAT-related motifs for interorganellar contact. Contact. 2021;4:1–11. doi: 10.1177/25152564211012246 34036242 PMC7610837

[34] Nishimura Y, Hayashi M, Inada H, et al. Molecular cloning and characterization of mammalian homologues of vesicle-associated membrane protein-associated (VAMP-associated) proteins. Biochem Biophys Res Commun. 1999;254(1):21–26. doi: 10.1006/bbrc.1998.9876 9920726

[35] Obara CJ, Nixon-Abell J, Moore AS, et al. Motion of VAPB molecules reveals ER-mitochondria contact site subdomains. Nature. 2024;626(7997):169–176. doi: 10.1038/s41586-023-06956-y 38267577 PMC10830423

[36] Dong R, Saheki Y, Swarup S, et al. Endosome-ER contacts control actin nucleation and retromer function through VAP-dependent regulation of PI4P. Cell. 2016;166(2):408–423. doi: 10.1016/j.cell.2016.06.037 27419871 PMC4963242

[37] Grumati P, Morozzi G, Holper S, et al. Full length RTN3 regulates turnover of tubular endoplasmic reticulum via selective autophagy. Elife. 2017;6:e25555. doi: 10.7554/eLife.25555 28617241 PMC5517149

[38] Nthiga TM, Kumar Shrestha B, Sjottem E, et al. Calcoco1 acts with VAMP-associated proteins to mediate ER-phagy. Embo J. 2020;39(15):e103649. doi: 10.15252/embj.2019103649 32525583 PMC7396842

[39] Hoyer MJ, Capitanio C, Smith IR, et al. Combinatorial selective ER-phagy remodels the ER during neurogenesis. Nat Cell Biol. 2024;3:1–46. 10.1038/s41556-024-01356-4 PMC10940164 38429475

[40] Zhao D, Zou CX, Liu XM, et al. A UPR-induced soluble ER-phagy receptor acts with VAPs to confer ER stress resistance. Mol Cell. 2020;79(6):963–977. doi: 10.1016/j.molcel.2020.07.019 32735772

[41] Molinari M. Er remodelling by ER-phagy in neurogenesis. Nat Cell Biol. 2024;26(3):1–2. doi: 10.1038/s41556-024-01362-6 38429474

[42] Kuijpers M, Kochlamazashvili G, Stumpf A, et al. Neuronal autophagy regulates presynaptic neurotransmission by controlling the axonal endoplasmic reticulum. Neuron. 2021;109(2):299–313 e9. doi: 10.1016/j.neuron.2020.10.005 33157003 PMC7837115

[43] Zhao K, Ridgway ND. Oxysterol-binding protein-related protein 1L regulates cholesterol egress from the endo-lysosomal system. Cell Rep. 2017;19(9):1807–1818. doi: 10.1016/j.celrep.2017.05.028 28564600

[44] Ellgaard L, Molinari M, Helenius A. Setting the standards: quality control in the secretory pathway. Science. 1999;286(5446):1882–1888. doi: 10.1126/science.286.5446.1882 10583943

[45] Marzo L, Marijanovic Z, Browman D, et al. 4-hydroxytamoxifen leads to PrPSc clearance by conveying both PrPC and PrPSc to lysosomes independently of autophagy. J Cell Sci. 2013;126:1345–1354. doi: 10.1242/jcs.114801 23418355

[46] Satpute-Krishnan P, Ajinkya M, Bhat S, et al. Er stress-induced clearance of misfolded GPI-anchored proteins via the secretory pathway. Cell. 2014;158(3):522–533. doi: 10.1016/j.cell.2014.06.026 25083867 PMC4121523

[47] Zavodszky E, Hegde RS. Misfolded GPI-anchored proteins are escorted through the secretory pathway by ER-derived factors. Elife. 2019;8:e46740. doi: 10.7554/eLife.46740 31094677 PMC6541436

[48] Forrester A, De Leonibus C, Grumati P, et al. A selective ER-phagy exerts procollagen quality control via a calnexin-FAM134B complex. Embo J. 2019;38(2):e99847. doi: 10.15252/embj.201899847 30559329 PMC6331724

[49] Omari S, Makareeva E, Roberts-Pilgrim A, et al. Noncanonical autophagy at ER exit sites regulates procollagen turnover. Proc Natl Acad Sci U S A. 2018;115(43):E10099–E10108. doi: 10.1073/pnas.1814552115 30287488 PMC6205486

[50] Tafaleng EN, Li J, Wang Y, et al. Variants in autophagy genes MTMR12 and FAM134A are putative modifiers of the hepatic phenotype in alpha1-antitrypsin deficiency. Hepatology. 2024;80(4):859–871. doi: 10.1097/HEP.0000000000000865 38557779 PMC11407773

[51] Reggiori F, Molinari M. Er-phagy: mechanisms, regulation and diseases connected to the lysosomal clearance of the endoplasmic reticulum. Physiol Rev. 2022;102(3):1393–1448. doi: 10.1152/physrev.00038.2021 35188422 PMC9126229

[52] Merulla J, Solda T, Molinari M. A novel UGGT1 and p97-dependent checkpoint for native ectodomains with ionizable intramembrane residue. Mol Biol Cell. 2015;26(8):1532–1542. doi: 10.1091/mbc.E14-12-1615 25694454 PMC4395132

[53] Fumagalli F, Noack J, Bergmann TJ, et al. Translocon component Sec62 acts in endoplasmic reticulum turnover during stress recovery. Nat Cell Biol. 2016;18(11):1173–1184. doi: 10.1038/ncb3423 27749824

[54] Rudinskiy M, Molinari M. Tandem fluorescent halo-GFP reporter for quantitative and time-resolved monitoring of organelle and protein delivery to lysosomes. Autophagy Rep. 2022;1(1):187–191. doi: 10.1080/27694127.2022.2061679 40396051 PMC11864615

[55] Rudinskiy M, Bergmann TJ, Molinari M. Quantitative and time-resolved monitoring of organelle and protein delivery to the lysosome with a tandem fluorescent halo-GFP reporter. Mol Biol Cell. 2022;33(6):1–13. doi: 10.1091/mbc.E21-10-0526 PMC9265146 35108065

